# Common genetic polymorphisms in pre-microRNAs and risk of bladder cancer

**DOI:** 10.1186/s12957-015-0683-6

**Published:** 2015-10-13

**Authors:** Shi Deng, Wei Wang, Xiang Li, Peng Zhang

**Affiliations:** Department of Urology, West China Hospital, Sichuan University, 37# Guoxuexiang Street, Chengdu, 610041 PR China; Department of Pathology, West China Second University Hospital, Chengdu, PR China

**Keywords:** Bladder tumor, SNPs, Cancer risk

## Abstract

**Background:**

At present, inconsistent association between single nucleotide polymorphism (SNP) in pre-miRNAs (*hsa-mir-196a2* rs11614913 C/T, *hsa-mir-499* rs3746444 A/G, and *hsa-mir-146a* rs2910164 C/G) and bladder cancer were obtained in limited studies. We performed a case–control study to test whether these three common polymorphisms are associated with bladder cancer. One hundred fifty-nine patients affected by bladder cancer and 298 unrelated healthy subjects were enrolled in the study.

**Methods:**

Using polymerase chain reaction–restriction fragment length polymorphism assay (PCR–RFLP), genotypes of these three SNPs were determined, and their associations with bladder cancer, as well as with clinic pathological factors, and tumor progression were analyzed.

**Results:**

No association between bladder cancer risk and variant allele of *hsa-mir-196a2* rs11614913 C/T, *hsa-mir-499* rs3746444 A/G, or *hsa-mir-146a* rs2910164 C/G was observed. Heterozygous genotype (CT genotype) of rs11614913 was associated with a significantly decreased bladder cancer risk (*P* = 0.004, OR = 0.56, 95 % CI = 0.38–0.83). Further stratified analyses showed that rs2910164 is associated with the tumor stage in a recessive model and with metastasis in a dominant model (*P* = 0.012, OR = 0.20, 95 % CI = 0.05–0.72 and *P* = 0.04, OR = 2.63, 95 % CI = 1.03–6.67, respectively). No association between *hsa-mir-499* rs3746444 A/G and bladder cancer was observed.

**Conclusions:**

Our results suggested *hsa-mir-196a2* rs11614913 C/T is associated with a significantly decreased risk of bladder cancer and *hsa-mir-146a* rs2910164 GG genotype is associated with clinical stage and metastasis in bladder cancer.

## Background

As the most common malignant tumor of the urinary tract, it is estimated that there were 74,690 new cases and 56,390 patients died from bladder cancer in United States in 2014 [1]. Tobacco smoking and chemical carcinogens are the two well-established external environmental factors of bladder cancer [2]. Besides, accumulating evidence indicates that inappropriate diet and lifestyle may have a relationship with bladder cancer [3]. These factors could be an explanation for the rising morbidity of bladder cancer all over the world. It is worth noting that the majority of exposed individuals do not suffer from bladder cancer in their lifetime. This phenomenon suggested that other causes, such as the genetic factor may also play a role in bladder carcinogenesis.

MicroRNAs (miRNAs) are a class of ancient, non-coding, and single-stranded molecules that participate in transcriptional and translational regulation by pairing with target mRNAs [4]. Although represents only 1–4 % in the human genome, a single miRNA can regulate approximately 200 target mRNAs [5]. This means that miRNAs may play critical roles in a diverse range of biological processes, such as cell proliferation, tissue differentiation, embryonic development, and apoptosis [5, 6]. Mutation of miRNAs and dysregulation of miRNAs and their targets genes have been observed in various diseases and several reports have demonstrated that miRNA plays an important role during carcinogenesis [7, 8].

Single nucleotide polymorphism (SNP) is the most common type of genetic variation that usually appears in miRNA coding genes, miRNA biogenesis genes, or in the miRNA-binding sites of mRNAs. The variations may alter the expression and function of miRNA, which may relate to the tumorigenesis [9]. Recently, two studies analyzed the relationship between common SNPs in pre-miRNAs and urinary bladder cancer [10]. While one obtained the negative results, the other indicates that miR-146a rs2910164 plays an important role in bladder cancer. In addition, they have associated the C allele with a significantly decreased risk and recurrence of bladder cancer [11]. To clarify the inconclusive results of previous studies, we explored the association between these three SNPs (rs2910164, rs11614913, and rs3746444) in pre-miRNAs (*hsa-mir-146a*, *hsa-mir-196a2*, and *hsa-mir-499*, respectively) and the risk of bladder cancer in a population of southwest China.

## Methods

### Patients and controls

All participants were form the Han population living in Sichuan Province of China and examined at the Department of urology, West China Hospital of Sichuan University. This study was approved by the Investigation and Ethics Committee of the West China Hospital of Sichuan University. Details were told to all participants and written informed consent was signed after the interview. The present study enrolled 159 unrelated patients with bladder cancer (124 men and 35 women, mean age: 64.09 ± 12.31) from the West China Hospital of Sichuan University between 2009 and 2012. The clinical diagnosis of bladder cancer was based on histopathologically confirmed specimens. Two hundred ninety-eight unrelated healthy individuals (140 men and 158 women, mean age: 60.21 ± 11.32) from a routine health survey were enrolled as controls. Patients who previously had cancer, radiotherapy and chemotherapy, or metastasized cancer from other or unknown origins were excluded. Control subjects were genetically unrelated individuals, and those with history of bladder cancer or other type of illness were excluded in this study.

### Genotyping

Three SNPs, including *hsa-mir-196a2* rs11614913 C/T, *hsa-mir-499* rs3746444 A/G, and *hsa-mir-146a* rs2910164 C/G were genotyped by the PCR–RFLP method. The instruction manual details that 300 μl of EDTA-anticoagulated peripheral blood samples should be collected from the patients and that their genomic DNA be isolated by a DNA isolation kit from Bioteke (Peking, China).

PIRA PCR designer (http://primer1.soton.ac.uk/primer2.html) was used to establish primers. The primers are used for amplification of these three SNPs and their specific restriction enzyme was shown in Table 1. The PCR reactions mixture was consisted of 2.0 μl 10× PCR buffer, 1.5 mmol/L MgCl_2_, 0.2 mmol/L dNTPs, 0.4 μmol/L each primer, 100 ng of genomic DNA and 1 U of *Taq* DNA polymerase. Briefly, cycling parameters were summarized as follows: 94 °C for 2 min; 30 cycles at 94 °C for 30 s; 30 s with different temperature parameters: 62 °C for rs11614913, 66 °C for rs3746444 and 58 °C for rs2910164; 45 s at 72 °C and the last extension procedure at 72 °C for 7 min. PCR–RFLP assay was used to obtained the genotypes of the three SNPs: rs11614913 C/T, rs3746444 A/G, rs2910164 C/G [12].

**Table 1:** Primers and restriction enzyme used for analysis of these three SNPS

SNPs	Primers (5′–3′)	Products (bp)	Restriction enzyme
*hsa-mir-196a2* rs11614913 C/T	F: CCCCTTCCCTTCTCCTCCAGATA	149	*MspI*
	R: CGAAAACCGACTGATGTAACTCCG		
*hsa-mir-499* rs3746444 A/G	F: CAAAGTCTTCACTTCCCTGCCA	146	*BclI*
	R: GATGTTTAACTCCTCTCCACGTGATC		
*hsa-mir-146a* rs2910164 C/G	F: CATGGGTTGTGTCAGTGTCAGAGCT	147	*SacI*
	R: TGCCTTCTGTCTCCAGTCTTCCAA		

Two researchers, who were irrelevant for this study, viewed the results independently. If there is no consensus, repeated assays would be required. Besides, 10 % duplicated samples were randomly selected to perform for the three SNPs, with a concordance rate of 100 %.

### Statistical analysis

Genotypic frequencies of the observed SNPs were available by directly counting and the *χ*^2^ test was used to evaluate the Hardy-Weinberg equilibrium. Data were carried out by using computer software SPSS (SPSS Inc., Chicago, IL, USA) and odds ratio (OR) and respective 95 % confidence intervals (95 % CI) were reported to compare the differences between the two groups. Genotypic association tests were conducted by using SNPstats, including codominant, dominant, recessive, overdominant, and log-additive genetic models [13].

## Results and discussion

Genotyping for all the three SNPs were performed in 159 bladder cancer patients and 298 control subjects. As expected, the results showed genotype frequencies for these three SNPs in control subjects were consistent with those from the Hardy-Weinberg equilibrium. Allele frequencies of rs11614913 C/T, rs3746444 A/G, and rs2910164 C/G in bladder cancer patients and controls are summarized in Table 2. As shown in Table 2, no significant difference for the allele frequencies of these three SNPs was observed between bladder cancer patients and control subjects (*P* = 0.976 for rs11614913, *P* = 0.348 for rs3746444, and *P* = 0.417 for rs2910164). Results of genotypic association tests performed by SNPstats are shown in Table 3. As shown in Table 3, significant association was identified for rs11614913 in an overdominant model. Compared with the combined homozygous genotypes (TT/CC genotypes), a significant decrease in bladder cancer risk was associated with the heterozygous genotype (CT genotype) of rs11614913 (*P* = 0.004, OR = 0.56, 95 % CI = 0.38–0.83). No association between the rs3746444 and rs2910164 genotypes and bladder cancer risk was observed in codominant, dominant, recessive, overdominant, or log-additive genetic model. These results suggest that the mir-196a2 rs11614913 C/T, but not the mir-499 rs3746444 A/G or the mir146a2 rs2910164 C/G, was associated with bladder cancer in the Chinese population studied. This indicates that polymorphisms in pre-microRNAs may play a role in the pathogenesis of bladder cancer.

**Table 2:** Allele frequencies of selected SNPs in pre-microRNAs among bladder cancer patients and controls

SNP	Allele	Patients	Controls	OR (95 % CI)	*P*
		*n* = 159 (%)	*n* = 298 (%)		
mir-196a2 rs11614913 C/T	C	148 (46.5)	278 (46.6)	1.00 (reference)	0.976
	T	170 (52.4)	318 (53.4)	0.996 (0.758–1.308)	
mir-499 rs3746444 A/G	A	259 (81.4)	500 (83.9)	1.00 (reference)	0.348
	G	59 (18.6)	96 (16.1)	1.186 (0.83–1.696)	
mir-146a rs2910164 G/C	C	193 (60.7)	378 (63.4)	1.00 (reference)	0.417
	G	125 (39.3)	218 (36.6)	1.123 (0.849–1.486)	

*n* number of individuals

**Table 3:** Genotype frequencies of selected SNPs in pre-microRNAs among bladder cancer patients and controls and their association with bladder cancer risk

Genetic model	Genotype	Patients	Controls	Logistic regression^a^	
		*n* = 159 (%)	*n* = 298 (%)	OR (95 % CI)	*P*
mir-196a2 rs11614913 C/T					
Codominant	T/T	52 (32.7 %)	76 (25.5 %)	1	0.015
	C/T	66 (41.5 %)	166 (55.7 %)	*0.58 (0.37–0.92)*	
	C/C	41 (25.8 %)	56 (18.8 %)	1.08 (0.63–1.82)	
Dominant	T/T	52 (32.7 %)	76 (25.5 %)	1	0.1
	C/T-C/C	107 (67.3 %)	222 (74.5 %)	0.70 (0.46–1.08)	
Recessive	T/T-C/T	118 (74.2 %)	242 (81.2 %)	1	0.085
	C/C	41 (25.8 %)	56 (18.8 %)	1.49 (0.95–2.38)	
Overdominant	T/T-C/C	93 (58.5 %)	132 (44.3 %)	1	*0.004*
	C/T	66 (41.5 %)	166 (55.7 %)	*0.56 (0.38–0.83)*	
Log-additive	—	—	—	1.00 (0.76–1.32)	0.98
mir-499 rs3746444 G/A					
Codominant	A/A	107 (67.3 %)	216 (72.5 %)	1	0.44
	G/A	45 (28.3 %)	68 (22.8 %)	1.33 (0.86–2.08)	
	G/G	7 (4.4 %)	14 (4.7 %)	0.99 (0.39–2.53)	
Dominant	A/A	107 (67.3 %)	216 (72.5 %)	1	0.25
	G/A-G/G	52 (32.7 %)	82 (27.5 %)	1.28 (0.84–1.96)	
Recessive	A/A-G/A	152 (95.6 %)	284 (95.3 %)	1	0.89
	G/G	7 (4.4 %)	14 (4.7 %)	0.93 (0.37–2.38)	
Overdominant	A/A-G/G	114 (71.7 %)	230 (77.2 %)	1	0.2
	G/A	45 (28.3 %)	68 (22.8 %)	1.33 (0.86–2.08)	
Log-additive	—	—	—	0.86 (0.61–1.20)	0.38
mir146a rs2910164 C/G					
Codominant	C/C	60 (37.7 %)	112 (37.6 %)	1	0.2
	C/G	73 (45.9 %)	154 (51.7 %)	0.88 (0.58–1.35)	
	G/G	26 (16.4 %)	32 (10.7 %)	1.52 (0.83–2.78)	
Dominant	C/C	60 (37.7 %)	112 (37.6 %)	1	0.97
	C/G-G/G	99 (62.3 %)	186 (62.4 %)	0.99(0.67–1.47)	
Recessive	C/C-C/G	133 (83.7 %)	266 (89.3 %)	1	0.091
	G/G	26 (16.4 %)	32 (10.7 %)	1.61 (0.93–2.86)	
Overdominant	C/C-G/G	86 (54.1 %)	144 (48.3 %)	1	0.24
	C/G	73 (45.9 %)	154 (51.7 %)	0.79 (0.54–1.16)	
Log-additive	—	—	—	0.88 (0.66–1.18)	0.4

^a^Adjusted by gender, age, smoking status, tumor stage, recurrence, and metastasis. Italicized values indicated a significant difference at the 5 % level

*n* number of individuals

Further analysis stratified bladder cancer patients’ characteristics including age, gender, smoking status, tumor stage, tumor grade, recurrence, and metastasis. As shown in Table 4, rs2910164 was found to be associated with the tumor stage in a recessive model (*P* = 0.012, OR = 0.20, 95 % CI = 0.05–0.72). Table 5 shows that rs2910164 is associated with metastasis in a dominant model (*P* = 0.04, OR = 2.63, 95 % CI = 1.03–6.67). No association was observed between these three SNPs and bladder cancer patients’ age, gender, smoking status, tumor grade, or recurrence (data not shown).

**Table 4:** Association between the selected SNPs in pre-microRNAs and tumor stage

Genetic model	Genotype	Invasive	Superficial	Logistic regression^a^	
		*n* = 84	*n* = 75	OR (95 % CI)	*P*
mir-196a2 rs11614913 C/T					
Codominant	T/T	27 (32.1 %)	25 (33.3 %)	1	0.68
	C/T	33 (39.3 %)	33 (44 %)	0.63 (0.21–1.86)	
	C/C	24 (28.6 %)	17 (22.7 %)	1.45 (0.43–5.00)	
Dominant	T/T	27 (32.1 %)	25 (33.3 %)	1	0.39
	C/T-C/C	57 (67.9 %)	50 (66.7 %)	0.65 (0.24–1.74)	
Recessive	T/T-C/T	60 (71.4 %)	58 (77.3 %)	1	0.83
	C/C	24 (28.6 %)	17 (22.7 %)	1.12 (0.39–3.23)	
Overdominant	T/T-C/C	51 (60.7 %)	42 (56 %)	1	0.52
	C/T	33 (39.3 %)	33 (44 %)	0.74 (0.28–1.91)	
Log-additive	—	—	—	0.82 (0.45–1.49)	0.51
mir-499 rs3746444 G/A					
Codominant	A/A	52 (61.9 %)	55 (73.3 %)	1	0.25
	G/A	28 (33.3 %)	17 (22.7 %)	1.96 (0.67–5.88)	
	G/G	4 (4.8 %)	3 (4 %)	3.70 (0.50–25.00)	
Dominant	A/A	52 (61.9 %)	55 (73.3 %)	1	0.12
	G/A-G/G	32 (38.1 %)	20 (26.7 %)	2.22 (0.81–5.88)	
Recessive	A/A-A/A	80 (95.2 %)	72 (96 %)	1	0.26
	G/G	4 (4.8 %)	3 (4 %)	3.13 (0.44–25.00)	
Overdominant	A/A-G/G	56 (66.7 %)	58 (77.3 %)	1	0.27
	G/A	28 (33.3 %)	17 (22.7 %)	1.82 (0.63–0.92)	
Log-additive	—	—	—	0.51 (0.23–1.13)	0.094
mir146a rs2910164 C/G					
Codominant	C/C	30 (35.7 %)	30 (40 %)	1	0.034
	C/G	45 (53.6 %)	28 (37.3 %)	1.41 (0.50–4.00)	
	G/G	9 (10.7 %)	17 (22.7 %)	0.24 (0.06–1.01)	
Dominant	C/C	30 (35.7 %)	30 (40 %)	1	0.84
	C/G-G/G	54 (64.3 %)	45 (60 %)	1.11 (0.42–2.89)	
Recessive	C/C-C/G	75 (89.3 %)	58 (77.3 %)	1	*0.012*
	G/G	9 (10.7 %)	17 (22.7 %)	*0.20 (0.05–0.72)*	
Overdominant	C/C-G/G	39 (46.4 %)	47 (62.7 %)	1	0.097
	C/G	45 (53.6 %)	28 (37.3 %)	2.17 (0.86–5.56)	
Log-additive	—	—	—	1.66 (0.84–3.31)	0.14

^a^Adjusted by gender, age, smoking status, tumor stage, recurrence, and metastasis. Italicized values indicated a significant difference at the 5 % level

*n* number of individuals

**Table 5:** Association between the selected SNPs in pre-microRNAs and metastasis

Genetic model	Genotype	Metastasis	No metastasis	Logistic regression^a^	
		*n* = 132	*n* = 27	OR (95 % CI)	*P*
mir-196a2 rs11614913 C/T					
Codominant	T/T	40 (30.3 %)	12 (44.4 %)	1	0.28
	C/T	56 (42.4 %)	10 (37 %)	1.33 (0.47–3.85)	
	C/C	36 (27.3 %)	5 (18.5 %)	2.63 (0.77–9.09)	
Dominant	T/T	40 (30.3 %)	12 (44.4 %)	1	0.23
	C/T-C/C	92 (69.7 %)	15 (55.6 %)	1.75 (0.70–4.35)	
Recessive	T/T-C/T	96 (72.7 %)	22 (81.5 %)	1	0.13
	C/C	36 (27.3 %)	5 (18.5 %)	2.33 (0.74–7.14)	
Overdominant	T/T-C/C	76 (57.6 %)	17 (63 %)	1	0.87
	C/T	56 (42.4 %)	10 (37 %)	1.08 (0.42–2.81)	
Log-additive	—	—	—	0.63 (0.35–1.14)	0.12
mir-499 rs3746444 G/A					
Codominant	A/A	89 (67.4 %)	18 (66.7 %)	1	0.37
	G/A	38 (28.8 %)	7 (25.9 %)	1.72 (0.60–5.00)	
	G/G	5 (3.8 %)	2 (7.4 %)	0.46 (0.06–3.45)	
Dominant	A/A	89 (67.4 %)	18 (66.7 %)	1	0.45
	G/A-G/G	43 (32.6 %)	9 (33.3 %)	0.69 (0.26–1.86)	
Recessive	A/A-G/A	127 (96.2 %)	25 (92.6 %)	1	0.35
	G/G	5 (3.8 %)	2 (7.4 %)	0.37 (0.05–2.70)	
Overdominant	A/A-G/G	94 (71.2 %)	20 (74.1 %)	1	0.23
	G/A	38 (28.8 %)	7 (25.9 %)	1.85 (0.65–5.26)	
Log-additive	—	—	—	0.89 (0.39–2.04)	0.78
mir146a rs2910164 C/G					
Codominant	C/C	46 (34.9 %)	14 (51.9 %)	1	0.081
	C/G	62 (47 %)	11 (40.7 %)	2.27 (0.83–6.25)	
	G/G	24 (18.2 %)	2 (7.4 %)	5.00 (0.86–25.00)	
Dominant	C/G	46 (34.9 %)	14 (51.9 %)	1	*0.04*
	C/G-G/G	86 (65.2 %)	13 (48.1 %)	*2.63 (1.03–6.67)*	
Recessive	C/G-C/G	108 (81.8 %)	25 (92.6 %)	1	0.12
	G/G	24 (18.2 %)	2 (7.4 %)	3.33 (0.62–16.67)	
Overdominant	C/C-G/G	70 (53 %)	16 (59.3 %)	1	0.31
	C/G	62 (47 %)	11 (40.7 %)	1.61 (0.63–4.17)	
Log-additive	—	—	—	*0.45 (0.21–0.93)*	*0.025*

^a^Adjusted by gender, age, smoking status, tumor stage, recurrence, and metastasis. Italicized values indicated a significant difference at the 5 % level

*n* number of individuals

In the present study, the association between SNPs in pre-miRNAs and bladder cancer, as well as with patients’ characteristics, such as age, gender, tumor grade, tumor stage, smoking status, recurrence, and metastasis were analyzed. Our results revealed that *hsa-mir-196a2* rs11614913 C/T is associated with bladder cancer in an overdominant model. Similarly, the *hsa-mir-146a* rs2910164 is associated with the tumor stage in recessive model and with metastasis in dominant model, respectively. Hsa-mir-499 rs3746444, however, is not associated with bladder cancer or any of the patients’ characteristics.

The miRNAs were composed of 21 to 24 nucleotides which can provide sequence specificity to RNA silencing pathway by pairing with target mRNAs [14]. Myriad evidence has indicated that miRNA is involved in diversity of biological process and many human genes are under its regulation, as well as some well-known tumor suppressor genes [5, 15]. Nowadays, the roles of altered miRNA expression in various diseases have been well established, including malignant cancer [7]. Gottardo and his colleagues analyzed the expression profile of miRNAs in bladder cancer and found that some miRNAs are significantly deregulated in bladder cancer compared with normal bladder mucosa, suggesting the involvement of these genes in the development and progression of bladder cancer [16]. Additionally, several additional miRNA-related SNPs have been demonstrated to be associated with bladder cancer risk, this means that miRNA-related SNPs are involved in mechanism of bladder carcinogenesis [17].

Emerging studies have reported the association between the *hsa-miR-196a2* rs11614913 polymorphism and susceptibility to several types of cancers, including hepatocellular carcinoma, gastric cancer, and breast cancer [18–20]. MiR-196a is one of the mature products encoded by miR196a2 gene, and miR-196a* which contains the SNP rs11614913 is the other mature product [20]. Hoffman et al. showed relative to empty vector control, the expression of mature miR-196a is increased in breast cancer cells both transfected with pre-miR-196a-C or miR-196a-T. Moreover, levels of the mature miR-196a were more than double in cells transfected with pre-miR-196a-C than those with miR-196a-T [20]. This finding suggested that SNP rs11614913 can affect the processing of the pre-miRNA into its mature, functional form, and this polymorphism might be a risk factor of tumorigenesis. This present study’s findings reveal that rs11614913 is associated with bladder cancer in an overdominant model, and the heterozygous genotype (CT genotype) was associated with decrease bladder cancer risk.

The rs2910164 is a common G/C polymorphism which resided at position +60 relative to the first nucleotide of pre-miR-146a [21]. Compared with the G allele, the Gibbs free was lower for rs2910164 C allele, suggesting that the secondary structure was less stable for the C allele. Genetic alterations might affect the expression and function of mature miR-146a [11]. By now, the susceptibility for carcinogenesis caused by the rs2910164 has been demonstrated in a series of studies. Jazdzewski et al*.* reported that the rs2910164 CC genotype is associated with lower risk in papillary thyroid carcinoma [21]. Studies in hepatocellular carcinoma, cervical squamous cell carcinoma, and bladder cancer also illustrate analogous results [11, 22, 12]. In vitro, Wang et al*.* found miR-146a can decrease bladder tumor cell proliferation and that the expression level of miR-146a is higher in rs2910164 C allele than G allele [11]. All of these results demonstrated that the C allele may act in a protective role and is more likely to exercise an inhibiting effect on the tumorigenesis. But the studies conducted in the breast/ovarian cancer showed an antithetical conclusion [15]. This diversity might be a result of organ-specific characteristics of rs2910164 and reflected the detailed mechanism of SNPs on cancer is not fully understood yet.

Invasive and metastatic nature was the main characteristic of malignance tumor, which was the primary cause of death for cancer patients [23]. Although exact molecular mechanisms remain unclear, several studies indicate that constitutive nuclear factor-κB (NF-κB) activity could promote the invasion and migration of human cancers [24, 25]. Known as the target of miR-146a, interleukin 1 receptor-associated kinase 1 (IRAK-1) plays an important role in activation of NF-κB [26, 27]. It has been reported that tumor cells would cease to be capable of invasion and migration if there was a downregulation of IRAK-1 induced by miR-146a [26, 28, 29]. In addition, cell movement mode was also believed to be a primary event in invasion and metastasis [30]. WASP family verprolin homologous protein 2 (WASF2) is involved in cell movement mode and several studies have confirmed WASF2 could promote tumor progression [30–33]. Recently, Yao found mir-146a can suppress the migration and invasion of gastric cancer cells by reduction of WASF2 expression [34]. Taken together, the above results suggest that mir-146a can repress tumor invasions and metastases in various ways.

In the present study, we found that SNP rs2910164 is associated with the tumor stage in a recessive model, with GG genotype frequency being significantly lower in the group with invasive bladder cancer. Moreover, allele G carriers (CG/GG genotype) are associated with metastasis in a dominant model. As for bladder cancer, 70 % patients belong to superficial cases [35]. Although the comprehensive treatment such as transurethral resection and postoperative intravesical chemotherapy are known as the gold standard nowadays, the progression rate within 5 years is still high [36]. Our study demonstrates that miR-146a rs2910164 is related to tumor stage and metastasis of bladder cancer. These findings reveal more clues about these SNPs of pre-miRNAs in the carcinogenesis of bladder cancer and may provide more information about tumor progression that has not yet been observed during an initial diagnosis.

## Conclusions

In conclusion, we found that hsa-mir-196a2 rs11614913 C/T is associated with a significantly decreased risk of bladder cancer in an overdominant model. In addition, hsa-mir-146a rs2910164 GG genotype is associated with clinical stage in a recessive model and CG/GG genotype is associated with metastasis in a dominant model. It is well known that the genetic polymorphism may execute a different effect among different racial or ethnic populations [37]. Hence, future large-scale investigations in different population should be performed to validate our results among other races and ethnicities.
